# Supplementary material for: Optical aberrations following implantation of multifocal intraocular lenses: a systematic review and meta-analysis protocol
Source: BMJ Open. 2022 Aug 18;12(8):e059350. doi: 10.1136/bmjopen-2021-059350 (PMC9394190; doi:10.1136/bmjopen-2021-059350)
Supplement: Supplementary data [file bmjopen-2021-059350supp001.pdf]

Ovid Medline

Search Lines	Search Terms
1	exp "Optics and Photonics"/
2	(optic* or photonic*).mp.
3	1 or 2
4	exp Refractive Errors/ or exp Refraction, Ocular/ or exp Astigmatism/ or exp Myopia/ or exp Visual Acuity/
5	(aberrat* or diffract* or refract* or HOA).mp.
6	4 or 5
7	exp Lenses, Intraocular/
8	(intraocular lens or Intra-ocular lens or intra ocular lens or IOL or IOLs or lens prosthes* or artificial lens).mp.
9	7 or 8
10	(multifocal or multi focal or multi-focal or bifocal or bi-focal or trifocal or tri-focal or hybrid).mp.
11	3 and 6 and 9 and 10

Ovid Embase

Search Lines	Search Terms
1	exp optics/
2	(optic* or photonic*).mp
3	1 or 2
4	exp eye refraction/
5	(aberrat* or diffract* or refract* or HOA).mp
6	4 or 5
7	exp lens implant/
8	(intraocular lens or Intra-ocular lens or intra ocular lens or IOL or IOLs or lens prosthes* or artificial lens).mp.
9	7 or 8
10	(multifocal or multi focal or multi-focal or bifocal or bi-focal or trifocal or tri-focal or hybrid).mp
11	3 and 6 and 9 and 10
