# Supplementary material for: Optical aberrations following implantation of multifocal intraocular lenses: a systematic review and meta-analysis protocol
Source: BMJ Open. 2022 Aug 18;12(8):e059350. doi: 10.1136/bmjopen-2021-059350 (PMC9394190; doi:10.1136/bmjopen-2021-059350)
Supplement: Author's manuscript [file bmjopen-2021-059350.draft_revisions.pdf]

BMJ Open is committed to open peer review. As part of this commitment we make the peer review history of every article we publish publicly available.

When an article is published we post the peer reviewers' comments and the authors' responses online. We also post the versions of the paper that were used during peer review. These are the versions that the peer review comments apply to.

The versions of the paper that follow are the versions that were submitted during the peer review process. They are not the versions of record or the final published versions. They should not be cited or distributed as the published version of this manuscript.

BMJ Open is an open access journal and the full, final, typeset and author-corrected version of record of the manuscript is available on our site with no access controls, subscription charges or pay-per-view fees (http://bmjopen.bmj.com).

If you have any questions on BMJ Open's open peer review process please email info.bmjopen@bmj.com

BMJ Open

Optical aberrations following implantation of multifocal IOLs: a systematic review protocol

Journal:	*BMJ Open*
Manuscript ID	bmjopen-2021-059350
Article Type:	Protocol
Date Submitted by the Author:	27-Nov-2021
Complete List of Authors:	Henein, Christin; University College London Institute of Ophthalmology; NIHR Moorfields Biomedical Research Centre<br>Fang, Clarissa E H; Manchester Royal Eye Hospital<br>Bokre, Desta; University College London Institute of Ophthalmology<br>Khan, Maaz; University College London Institute of Ophthalmology<br>Adan, Ahmed; University College London Institute of Ophthalmology<br>Bouremel, Yann; University College London Institute of Ophthalmology<br>Nanavaty, Mayank; Sussex Eye Hospital; Brighton and Sussex Medical School
Keywords:	Cataract and refractive surgery < OPHTHALMOLOGY, Ophthalmology < SURGERY, OPHTHALMOLOGY

SCHOLARONE™
Manuscripts

Optical aberrations following implantation of multifocal IOLs: a systematic review protocol

Authors:

Christin Henein[1,2], Clarissa E.H. Fang[3], Desta Bokre[1], Maaz Khan[1], Ahmed Adan[1], Yann Bouremel[1], Mayank A. Nanavaty[4,5]

Affiliations

[1]UCL, Institute of Ophthalmology, London, UK
[2]NIHR Moorfields Biomedical Research Centre, London, UK
[3]Manchester Royal Eye Hospital, Manchester, UK
[4]Sussex Eye Hospital, University Hospitals Sussex NHS Foundation Trust, Eastern Road, Brighton, United Kingdom. BN2 5BF
[5]Brighton & Sussex Medical School, University of Sussex, Falmer, Brighton. United Kingdom. BN1 9PX

Corresponding author*

I, the Submitting Author has the right to grant and does grant on behalf of all authors of the Work (as defined in the below author licence), an exclusive licence and/or a non-exclusive licence for contributions from authors who are: i) UK Crown employees; ii) where BMJ has agreed a CC-BY licence shall apply, and/or iii) in accordance with the terms applicable for US Federal Government officers or employees acting as part of their official duties; on a worldwide, perpetual, irrevocable, royalty-free basis to BMJ Publishing Group Ltd ("BMJ") its licensees and where the relevant Journal is co-owned by BMJ to the co-owners of the Journal, to publish the Work in BMJ Open and any other BMJ products and to exploit all rights, as set out in our licence.

Authors contribution

MN conceived the idea for the review. CH, MK, AA and DB drafted and revised the protocol with suggestions from YB, CEHF and MN who reviewed the protocol and provided feedback on the draft. DB constructed the search.

Conflicts of interest

For peer review only

The authors declare no conflict of interest.

Data sharing statement

No additional data is available.

Patient and Public Involvement

Patients and public were not involved in the development of this protocol. The primary outcome of the review is patient-centred.

Acknowledgments

The authors would like to acknowledge the funding as mentioned below.

Funding

CH receives support from the National Institute for Health Research CL 2020-18-009

Email addresses:

c.henein@ucl.ac.uk, d.bokre@ucl.ac.uk, maaz.khan.20@ucl.ac.uk, ahmed.adan.20@ucl.ac.uk, y.bouremel@ucl.ac.uk, fangclarissa@gmail.com, mayank.nanavaty@nhs.net

For peer review only - http://bmjopen.bmj.com/site/about/guidelines.xhtml

Abstract

Introduction: Multifocal IOLs are used to restore vision at different focal distances. The technology of multifocal IOLs is continually advancing. Optical aberrations a property of lenses that causes spreading of light over a region resulting in a blurred or distorted image. This study aims to systematically review investigator measured and patient reported optical aberrations following implantation of multifocal intraocular lenses during phacoemulsification surgery to treat presbyopia in adults.

Methods and Analysis: We will conduct an electronic database search for randomized controlled trials, prospective non-randomized studies, observational studies in Ovid MEDLINE, Ovid EMBASE, Cochrane Central Register of Controlled Trials (CENTRAL), Web of Science, Scopus, and ClinicalTrials.gov. Eligibility criteria will include quantitative articles written in English and containing data on optical aberrations. Two independent reviewers will screen titles and abstracts and extract data from full texts, reporting outcomes according to Preferred Reporting Items for Systematic Reviews and Meta-Analyses (PRISMA) guidelines. Data extraction of key characteristics will be completed using customized forms. Methodological quality will be assessed using Cochrane Handbook 6.2.

Ethics and Dissemination Ethics approval is not required for this review, as it will only include published data. Findings will be published in a peer-reviewed journal and disseminated across ophthalmic networks. We anticipate that the findings of this work will be of interest to multiple stakeholders: people who have undergone cataract surgery, eye health professionals, ophthalmic surgeons, device manufacturers and policy makers. It will also inform researchers to where there are gaps in evidence and identify areas for future research.

Systematic review registration number: PROSPERO CRD42021271050

Keywords: Optics, aberration, intraocular lens, multifocal

Article Summary

Strengths and Limitations

For peer review only

For peer review only - http://bmjopen.bmj.com/site/about/guidelines.xhtml

- This systematic review protocol follows the Preferred Reporting Items for Systematic Review and Meta-Analysis Protocols guidelines.
- This systematic review addresses a gap in the current evidence-base by providing a comprehensive assessment of reported optical aberrations following new and older generation multifocal IOL

Word count: 1507

Introduction

Traditional monofocal IOLs provide a single point of focus and toric monofocal lenses can correct astigmatism. Multifocal IOLs have multiple focal lengths. If they have 2 foci, they are called bifocal, three foci, they are trifocal. This enables the patient with a multifocal IOL to see both objects located at a distance or near to them. They are three different mechanisms to achieve this: the technology can be refractive, diffractive or combined. Moreover, toric multifocal lens also help to correct the problem of astigmatism that only toric monofocal lens can do[1].

Multifocal IOLs are used to restore vision at different focal distances. It is generally accepted they are good for distance and intermediate focal distances. According to the lens design they can be refractive, diffractive or combined. The technology of multifocal IOLs is continually advancing. Next generation IOLs include rotationally asymmetric segmented multifocal IOL, increase in the central area with the aim to improve reading acuity, improved pupil independence and increased depth of focus. Optical aberrations a property of lenses that causes spreading of light over a region resulting in a blurred or distorted image. Optical aberrations can present as symptoms of glare, holes and stars. This symptoms may limit the patient satisfaction achieved with these IOLs and is therefore an important patient-centred outcomes to quantify. Spherical aberrations significantly contribute to quality of retinal image and subjective refraction. Optical aberrations can be reported subjectively using questionnaires or measured objectively by wavefront aberrometry analysis. Contrast sensitivity can be a more useful/ objective tool to assess visual function. Recent reviews that compared multifocal with monofocal IOLs

For peer review only

100 reported outcomes on spectacle independence, visual acuity and quality of life[2,3]. To our knowledge this is the first review comparing different multifocal IOLs with optical aberrations as the primary outcome.

103 **Review aim:** We aim to systematically review investigator measured and patient reported optical aberrations following implantation of multifocal intraocular lenses during phacoemulsification surgery to treat presbyopia.

106 **Methods and analysis**

107 **Inclusion and exclusion criteria**

108 **Types of studies**

109 We will include randomised controlled trials (RCTs) and non-randomised interventional studies (retrospective or prospective studies). Observational studies will allow us to provide real-world estimates of reported optical aberrations.

112 **Types of participants**

113 We will include adults aged 18 years and above with presbyopia. We will exclude studies with participants with history of laser refractive surgery.

115 **Intervention(s)**

116 We will include small incision cataract extraction and multifocal lens implantation. All types of refractive and diffractive multifocal lenses will be included in this review.

118 **Comparator(s)**

119 We will include multifocal intraocular lens or alternative type of multifocal IOL as comparators such as diffractive, refractive and hybrid technologies.

121 **Outcomes**

For peer review only

For peer review only - http://bmjopen.bmj.com/site/about/guidelines.xhtml

Primary outcome

- Participant reported optical aberrations such as but not limited to glare and halos.

Secondary outcomes

- Measured optical aberrations with wavefront analysis
- Contrast sensitivity as measured by validated test
- Spectacle independence as determined by the participant or as determined by the investigator
- Uncorrected near vision acuity
- Uncorrected distance vision acuity
- Mean spherical equivalent within ±0.5D
- % of eyes seeing 20/20 or better for distance
- % of eyes seeing 20/40 or better for distance
- % of eyes seeing J5 or better for near vision
- YAG laser capsulotomy rates

Search strategy

In collaboration with an information specialist a comprehensive search strategy will be performed using a combination of controlled vocabulary and free text terms. Searches will be conducted in Ovid MEDLINE, Ovid EMBASE, Cochrane Central Register of Controlled Trials (CENTRAL), Web of Science, Scopus and ClinicalTrials.gov bibliographic databases. Other relevant sources will be searched such as reference lists of existing systematic reviews of multifocal IOLs. Please see appendix 1 for strategy syntax for Ovid Medline 1946 – March 2021 electronic database. We will download references identified in searches (electronic database and additional searches) into Endnote X9 reference management software and remove duplicate abstracts.

Study selection

For peer review only

146 The screening process will be undertaken using Endnote X9. Two review authors will independently
147 assess the titles and abstracts of records and exclude papers that do not meet eligibility criteria. We
148 will obtain the full text of the remaining papers, and at least two authors will assess the papers against
149 the inclusion criteria for the review to determine their eligibility for inclusion. Non-English language
150 papers will be excluded. The review authors will resolve disagreements through mediation with a third
151 reviewer.

152 **Data extraction**

153 Two review authors will extract data independently using Excel. We will pre-pilot the data extraction
154 template. We will resolve discrepancies by discussion. Two attempts will be made to contact trial
155 investigators for missing data. Data will be directly imported into Review Manager 5 (RevMan 5); and
156 the accuracy of the data import will be checked by one author.

157 We will collect the following information on study characteristics:

158 • Study design: parallel group RCT/within-person RCT/one or both eyes reported
159 • Participants: country, total number of participants, age, sex, inclusion and exclusion
160 criteria
161 • Intervention and comparator details: type of multifocal IOL, including number of people
162 (eyes) randomised to each group
163 • Primary and secondary outcomes as measured and reported in the trials
164 • Length of follow-up
165 • Date of publication
166 • Date multifocal IOL received market approval (FDA PMA, CE mark)
167 • Sample size
168 • Funding and conflicts of interest
169 • Trial registration, if available

For peer review only

For peer review only - http://bmjopen.bmj.com/site/about/guidelines.xhtml

Data synthesis

We will pool data using a random-effects model in RevMan 5. If there are fewer than three trials in a comparison we will use a fixed-effect model. If there is inconsistency between individual study results such that a pooled result may not be a good summary of the individual trial results — for example, the effects are in different directions or $I^2 > 50\%$ and $P < 0.1$ — we will not pool the data but will describe the pattern of the individual study results. If there is statistical heterogeneity we may pool the data if all the effect estimates are in the same direction, such that a pooled estimate would seem to provide a good summary of the individual trial results.

We will extract the following data from each included study for intervention and comparator groups separately.

- Number of events and number of participants on which outcome data collected for dichotomous variables
- Mean, standard deviation and number of participants on which outcome measured for continuous variables

For multi-arm studies we will use data relevant to our intervention and comparator groups. If two groups contain relevant data we will combine groups using the calculator within RevMan 5. If standard deviation is not available we will use information from confidence intervals and P values, where possible, to estimate it, using the RevMan 5 calculator[4].

Assessment of risk of bias in included studies

Two review authors will assess independently the risk of bias using Cochrane's 'Risk of bias' tool for assessing risk of bias in each included study according to the following domains selection bias, performance bias, detection bias, attrition bias and selective outcome reporting bias[5]. We will resolve disagreements by discussion. We will specifically consider and report on the following sources of bias. We will grade each domain as low risk of bias, high risk of bias or unclear (lack of information or uncertainty of potential for bias). We will attempt to contact trial investigators for clarification of parameters graded as 'unclear'.

For peer review only

Dealing with missing data

If possible, we will conduct an intention-to-treat (ITT) analysis. We will use imputed data if computed by the trial investigators using an appropriate method, but will not impute missing data ourselves. If ITT data are not available, we will do an available case analysis. This assumes that data are missing at random. We will assess whether this assumption is reasonable by collecting data from each included trial on the number of participants excluded or lost to follow-up and reasons for loss to follow-up by treatment group, if reported.

Assessment of heterogeneity and subgroup analysis

We will examine the overall characteristics of the studies, in particular the type of participants and types of interventions, to assess the extent to which the studies are similar enough to make pooling study results sensible. We will look at the forest plots of study results to see how consistent the results of the studies are, in particular looking at the size and direction of effects. We will calculate I^2 which is the percentage of the variability in effect estimates that is due to heterogeneity rather than sampling error. We will consider I^2 values over 50% to indicate substantial inconsistency but will also consider Chi^2 P value. As this may have low power when the number of studies are few we will consider $P < 0.1$ to indicate statistical significance of the Chi^2 test. If there are sufficient trials we will compare the effect of treatment in the following subgroups; diffractive, refractive and hybrid multifocal IOL and year of market approval.

Sensitivity analysis and assessment of reporting biases

We will examine the impact of excluding studies at high risk of bias in one or more domains. If there are 10 trials or more included in a meta-analysis, we will construct funnel plots and consider tests for asymmetry for assessment of publication bias, according to Chapter 8 of the Cochrane Handbook for Systematic Reviews of Interventions[6].

References

For peer review only

1. Salerno LC, Tiveron MC, Jr., Alió JL. Multifocal intraocular lenses: Types, outcomes, complications and how to solve them. *Taiwan J Ophthalmol.* 2017;7(4):179-184.
2. Khandelwal SS, Jun JJ, Mak SS, Booth MS, Shekelle P. Effectiveness of multifocal and monofocal intraocular lenses for cataract surgery and lens replacement: a systematic review and meta-analysis. *Graefe's Archive for Clinical and Experimental Ophthalmology.* 2018;257:863-875.
3. de Silva SR, Evans JR, Kirthi V, Ziaei M, Leyland M. Multifocal versus monofocal intraocular lenses after cataract extraction. *Cochrane Database Syst Rev.* 2016;12(12):Cd003169.
4. X. Wan WW, J. Liu, T. Tong. Estimating the sample mean and standard deviation from the sample size, median, range and/or interquartile range. *BMC Med Res Methodol.* 2014:135.
5. Higgins JP, Altman DG, Gøtzsche PC, et al. The Cochrane Collaboration's tool for assessing risk of bias in randomised trials. *Bmj.* 2011;343:d5928.
6. Higgins JPT TJ, Chandler J, Cumpston M, Li T, Page MJ, Welch VA (editors). Cochrane Handbook for Systematic Reviews of Interventions version 6.2 (updated February 2021). 2021.

For peer review only

For peer review only - http://bmjopen.bmj.com/site/about/guidelines.xhtml

Appendix 1. Search strategy

Ovid Medline 1946 – March 2021

Search Lines	Search Terms	Search Results
1	exp "Optics and Photonics"/	79373
2	(optic* or photonic*).mp.	476463
3	1 or 2	518769
4	exp Refractive Errors/ or exp Refraction, Ocular/ or exp Astigmatism/ or exp Myopia/ or exp Visual Acuity/	108238
5	(aberrat* or diffract* or refract* or HOA).mp.	461904
6	4 or 5	541199
7	exp Lenses, Intraocular/	15452
8	(intraocular lens or Intra-ocular lens or intra ocular lens or IOL or IOLs or lens prosthes* or artificial lens).mp.	18494
9	7 or 8	23720
10	(multifocal or multi focal or multi-focal or bifocal or bi-focal or trifocal or tri-focal or hybrid).mp.	218357
11	3 and 6 and 9 and 10	515

For peer review only

PRISMA-P (Preferred Reporting Items for Systematic review and Meta-Analysis Protocols) 2015 checklist: recommended items to address in a systematic review protocol*

Section and topic	Item No	Checklist item	Page Line
**ADMINISTRATIVE INFORMATION**			
Title:			
Identification	1a	Identify the report as a protocol of a systematic review	P1 L1
Update	1b	If the protocol is for an update of a previous systematic review, identify as such	P1 L1
Registration	2	If registered, provide the name of the registry (such as PROSPERO) and registration number	P3 L62
Authors:			
Contact	3a	Provide name, institutional affiliation, e-mail address of all protocol authors; provide physical mailing address of corresponding author	P2 L39
Contributions	3b	Describe contributions of protocol authors and identify the guarantor of the review	P1 L23
Amendments	4	If the protocol represents an amendment of a previously completed or published protocol, identify as such and list changes; otherwise, state plan for documenting important protocol amendments	NA
Support:			
Sources	5a	Indicate sources of financial or other support for the review	P2 L35
Sponsor	5b	Provide name for the review funder and/or sponsor	P2 L35
Role of sponsor or funder	5c	Describe roles of funder(s), sponsor(s), and/or institution(s), if any, in developing the protocol	NA
**INTRODUCTION**			
Rationale	6	Describe the rationale for the review in the context of what is already known	P4 L76
Objectives	7	Provide an explicit statement of the question(s) the review will address with reference to participants, interventions, comparators, and outcomes (PICO)	P4 L87
**METHODS**			
Eligibility criteria	8	Specify the study characteristics (such as PICO, study design, setting, time frame) and report characteristics (such as years considered, language, publication status) to be used as criteria for eligibility for the review	P4 L90
Information sources	9	Describe all intended information sources (such as electronic databases, contact with study authors, trial registers or other grey literature sources) with planned dates of coverage	P6 L122
Search strategy	10	Present draft of search strategy to be used for at least one electronic database, including planned limits, such that it could be repeated	P6 L22

For peer review only

Study records:			
Data management	11a	Describe the mechanism(s) that will be used to manage records and data throughout the review	P6 L136
Selection process	11b	State the process that will be used for selecting studies (such as two independent reviewers) through each phase of the review (that is, screening, eligibility and inclusion in meta-analysis)	P6 L132
Data collection process	11c	Describe planned method of extracting data from reports (such as piloting forms, done independently, in duplicate), any processes for obtaining and confirming data from investigators	P6 137
Data items	12	List and define all variables for which data will be sought (such as PICO items, funding sources), any pre-planned data assumptions and simplifications	P6 L141
Outcomes and prioritization	13	List and define all outcomes for which data will be sought, including prioritization of main and additional outcomes, with rationale	P5 L105
Risk of bias in individual studies	14	Describe anticipated methods for assessing risk of bias of individual studies, including whether this will be done at the outcome or study level, or both; state how this information will be used in data synthesis	P8 L147
Data synthesis	15a	Describe criteria under which study data will be quantitatively synthesised	P7 L154
	15b	If data are appropriate for quantitative synthesis, describe planned summary measures, methods of handling data and methods of combining data from studies, including any planned exploration of consistency (such as I^2, Kendall's τ)	P7 L154
	15c	Describe any proposed additional analyses (such as sensitivity or subgroup analyses, meta-regression)	P9 L208
	15d	If quantitative synthesis is not appropriate, describe the type of summary planned	P7 L159
Meta-bias(es)	16	Specify any planned assessment of meta-bias(es) (such as publication bias across studies, selective reporting within studies)	P9 L213
Confidence in cumulative evidence	17	Describe how the strength of the body of evidence will be assessed (such as GRADE)	P9 L217

*** It is strongly recommended that this checklist be read in conjunction with the PRISMA-P Explanation and Elaboration (cite when available) for important clarification on the items. Amendments to a review protocol should be tracked and dated. The copyright for PRISMA-P (including checklist) is held by the PRISMA-P Group and is distributed under a Creative Commons Attribution Licence 4.0.**

From: Shamseer L, Moher D, Clarke M, Ghersi D, Liberati A, Petticrew M, Shekelle P, Stewart L, PRISMA-P Group. Preferred reporting items for systematic review and meta-analysis protocols (PRISMA-P) 2015: elaboration and explanation. BMJ. 2015 Jan 2;349(jan02 1):g7647.

For peer review only

BMJ Open

Optical aberrations following implantation of multifocal intraocular lenses: a systematic review and meta-analysis protocol

Journal:	*BMJ Open*
Manuscript ID	bmjopen-2021-059350.R1
Article Type:	Protocol
Date Submitted by the Author:	24-Jun-2022
Complete List of Authors:	Henein, Christin; University College London Institute of Ophthalmology; NIHR Moorfields Biomedical Research Centre<br>Fang, Clarissa E H; Manchester Royal Eye Hospital<br>Bokre, Desta; University College London Institute of Ophthalmology<br>Khan, Maaz; University College London Institute of Ophthalmology<br>Adan, Ahmed; University College London Institute of Ophthalmology<br>Bouremel, Yann; University College London Institute of Ophthalmology<br>Nanavaty, Mayank; Sussex Eye Hospital; Brighton and Sussex Medical School
**Primary Subject Heading**:	Ophthalmology
Secondary Subject Heading:	Surgery, Evidence based practice, Ophthalmology
Keywords:	Cataract and refractive surgery < OPHTHALMOLOGY, Ophthalmology < SURGERY, OPHTHALMOLOGY

SCHOLARONE™
Manuscripts

Optical aberrations following implantation of multifocal intraocular lenses: a systematic review and meta-analysis protocol

Authors:

Christin Henein*[1,2], Clarissa E.H. Fang[3], Desta Bokre[1], Maaz Khan[1], Ahmed Adan[1], Yann Bouremel[1], Mayank A. Nanavaty[4,5]

*** Correspondence to Dr Christin Henein; c.henein@ucl.ac.uk**

Affiliations

[1]UCL, Institute of Ophthalmology, London, UK

[2]NIHR Moorfields Biomedical Research Centre, London, UK

[3]Manchester Royal Eye Hospital, Manchester, UK

[4]Sussex Eye Hospital, University Hospitals Sussex NHS Foundation Trust, Eastern Road, Brighton, United Kingdom. BN2 5BF

[5]Brighton & Sussex Medical School, University of Sussex, Falmer, Brighton. United Kingdom. BN1 9PX

Corresponding author*

I, the Submitting Author has the right to grant and does grant on behalf of all authors of the Work (as defined in the below author licence), an exclusive licence and/or a non-exclusive licence for contributions from authors who are: i) UK Crown employees; ii) where BMJ has agreed a CC-BY licence shall apply, and/or iii) in accordance with the terms applicable for US Federal Government officers or employees acting as part of their official duties; on a worldwide, perpetual, irrevocable, royalty-free basis to BMJ Publishing Group Ltd ("BMJ") its licensees and where the relevant Journal is co-owned by BMJ to the co-owners of the Journal, to publish the Work in BMJ Open and any other BMJ products and to exploit all rights, as set out in our licence.

Email addresses:

c.henein@ucl.ac.uk, d.bokre@ucl.ac.uk, maaz.khan.20@ucl.ac.uk, ahmed.adan.20@ucl.ac.uk, y.bouremel@ucl.ac.uk, fangclarissa@gmail.com, mayank.nanavaty@nhs.net

For peer review only

31

For peer review only

For peer review only - http://bmjopen.bmj.com/site/about/guidelines.xhtml

Abstract

Introduction: Multifocal IOLs are used to restore vision at different focal distances. The technology of multifocal IOLs is continually advancing. Optical aberrations a property of lenses that causes spreading of light over a region resulting in a blurred or distorted image. This study aims to systematically review investigator measured and patient reported optical aberrations following implantation of multifocal intraocular lenses during phacoemulsification surgery to treat presbyopia in adults.

Methods and Analysis: We will conduct an electronic database search for randomized controlled trials, prospective non-randomized studies, observational studies in Ovid MEDLINE, Ovid EMBASE, Cochrane Central Register of Controlled Trials (CENTRAL), Web of Science, Scopus, and ClinicalTrials.gov in March 2021. Eligibility criteria will include quantitative articles written in English and containing data on optical aberrations. Two independent reviewers will screen titles and abstracts and extract data from full texts, reporting outcomes according to Preferred Reporting Items for Systematic Reviews and Meta-Analyses (PRISMA) guidelines. Data extraction of key characteristics will be completed using customized forms. Methodological quality will be assessed using Cochrane Handbook 6.2.

Systematic review registration number: PROSPERO CRD42021271050

Keywords: Optics, aberration, intraocular lens, multifocal

Article Summary

Strengths and Limitations

- This systematic review protocol follows the Preferred Reporting Items for Systematic Review and Meta-Analysis Protocols guidelines.
- This systematic review addresses a gap in the current evidence-base by providing a comprehensive assessment of reported optical aberrations following new and older generation multifocal IOL

For peer review only

For peer review only - http://bmjopen.bmj.com/site/about/guidelines.xhtml

- There may be a paucity of RCTs comparing different multifocal IOLs limiting the number of paired wise meta-analysis that can be done.

Word count: 1507

Introduction

Multifocal intraocular lens (IOLs) have multiple focal lengths; if they have 2 foci, they are called bifocal, three foci, they are trifocal. This enables the patient with a multifocal IOL to see both objects located at a distance, intermediate distance or near to them. They are three different mechanisms to achieve this: the technology can be refractive, diffractive or combined. Moreover, toric multifocal lens also help to correct the problem of astigmatism [1].

Traditional monofocal IOLs provide a single point of focus. A newer enhanced monofocals and extended depth-of-focus (EDOF) IOLs which creates a single elongated focal point to enhance the depth of focus. For the purposes of this study we will assess optical aberrations following the implantation of different types of multifocal IOL and will exclude enhanced monofocal IOL as a well as EDOF IOLs.

It is generally accepted multifocal IOLS are good for distance and near focal distances. According to the lens design they can be refractive, diffractive or combined. The technology of multifocal IOLs is continually advancing. Next generation IOLs include rotationally asymmetric segmented multifocal IOL, increase in the central area with the aim to improve reading acuity and improved pupil independence.

Optical aberrations a property of lenses that causes spreading of light over a region resulting in a blurred or distorted image. Optical aberrations can present as symptoms of glare, holes and stars. This symptoms may limit the patient satisfaction achieved with these IOLs and is therefore an important patient-centred outcomes to quantify. Spherical aberrations significantly contribute to quality of retinal image and subjective refraction. Optical aberrations can be reported subjectively using questionnaires or measured objectively by wavefront aberrometry analysis. Contrast sensitivity can be a more useful/ objective tool to assess visual function. Recent reviews that compared multifocal with monofocal IOLs

For peer review only

reported outcomes on spectacle independence, visual acuity and quality of life [2, 3]. To our knowledge this is the first review comparing different multifocal IOLs with optical aberrations as the primary outcome.

Review aim: We aim to systematically review investigator measured and patient reported optical aberrations following implantation of multifocal intraocular lenses during phacoemulsification surgery to treat presbyopia.

Methods and analysis

Inclusion and exclusion criteria

Types of studies

We will include randomised controlled trials (RCTs) and non-randomised interventional studies (retrospective or prospective studies). Observational studies will allow us to provide real-world estimates of reported optical aberrations.

Types of participants

We will include adults undergoing cataract surgery and desiring correction for anticipated post-operative presbyopia. We will exclude studies with participants with history of laser refractive surgery.

Intervention(s)

We will include small incision cataract extraction and multifocal lens implantation. All types of refractive and diffractive multifocal lenses will be included in this review.

Comparator(s)

We will include multifocal intraocular lens or alternative type of multifocal IOL as comparators such as diffractive, refractive and hybrid technologies.

Outcomes

For peer review only - http://bmjopen.bmj.com/site/about/guidelines.xhtml

Primary outcome

- Participant reported optical aberrations such as but not limited to glare and halos.

Secondary outcomes

- Measured optical aberrations with wavefront analysis
- Contrast sensitivity as measured by validated test
- Spectacle independence as determined by the participant or as determined by the investigator
- Uncorrected near vision acuity
- Uncorrected distance vision acuity
- Uncorrected intermediate distance
- Mean spherical equivalent within ±0.5D
- % of eyes seeing 20/20 or better for distance
- % of eyes seeing 20/40 or better for distance
- % of eyes seeing J2 or better for near vision
- YAG laser capsulotomy rates

Search strategy

In collaboration with an information specialist a comprehensive search strategy will be performed using a combination of controlled vocabulary and free text terms. Searches will be conducted in Ovid MEDLINE, Ovid EMBASE, Cochrane Central Register of Controlled Trials (CENTRAL), Web of Science, Scopus and ClinicalTrials.gov bibliographic databases. Other relevant sources will be searched such as reference lists of existing systematic reviews of multifocal IOLs. Please see supplementary file 1 for strategy syntax for Ovid Medline 1946 – March 2021 electronic database. We will download references identified in searches (electronic database and additional searches) into Endnote X9 reference management software and remove duplicate abstracts.

Study selection

For peer review only

133 The screening process will be undertaken using Endnote X9. Two review authors will independently assess the titles and abstracts of records and exclude papers that do not meet eligibility criteria. We will obtain the full text of the remaining papers, and at least two authors will assess the papers against the inclusion criteria for the review to determine their eligibility for inclusion. Non-English language papers will be excluded. The review authors will resolve disagreements through mediation with a third reviewer.

139 **Data extraction**

140 Two review authors will extract data independently using Excel. We will pre-pilot the data extraction template. We will resolve discrepancies by discussion. Two attempts will be made to contact trial investigators for missing data. Data will be directly imported into Review Manager 5 (RevMan 5); and the accuracy of the data import will be checked by one author.

144 We will collect the following information on study characteristics:

145 • Study design: parallel group RCT/within-person RCT/one or both eyes reported

146 • Participants: country, total number of participants, age, sex, inclusion and exclusion criteria

148 • Intervention and comparator details: type of multifocal IOL, including number of people (eyes) randomised to each group

150 • Primary and secondary outcomes as measured and reported in the trials

151 • Length of follow-up

152 • Date of publication

153 • Date multifocal IOL received market approval (FDA PMA, CE mark)

154 • Sample size

155 • Funding and conflicts of interest

156 • Trial registration, if available

For peer review only - http://bmjopen.bmj.com/site/about/guidelines.xhtml

157 **Data synthesis**

158 We will pool data where there are at least two studies for a particular type of mIOL reporting the same
159 outcome. We will use a random-effects model in RevMan 5. But if there are fewer than three trials in a
160 comparison we will use a fixed-effect model. If there is inconsistency between individual study results
161 such that a pooled result may not be a good summary of the individual trial results — for example, the
162 effects are in different directions or $I^2 > 50\%$ and $P < 0.1$ — we will not pool the data but will describe
163 the pattern of the individual study results. If there is statistical heterogeneity we may pool the data if all
164 the effect estimates are in the same direction, such that a pooled estimate would seem to provide a
165 good summary of the individual trial results.

166 We will extract the following data from each included study for intervention and comparator groups
167 separately.

168 • Number of events and number of participants on which outcome data collected for
169 dichotomous variables

170 • Mean, standard deviation and number of participants on which outcome measured for
171 continuous variables

172 For multi-arm studies we will use data relevant to our intervention and comparator groups. If two
173 groups contain relevant data we will combine groups using the calculator within RevMan 5. If standard
174 deviation is not available we will use information from confidence intervals and P values, where
175 possible, to estimate it, using the RevMan 5 calculator [4].

176 For the primary outcome a power calculation will made using *metapower* package in R (rstudio.com)
177 to calculate the statistical power for meta-analysis based on Cohen's *d [5]*. We expect to find at least
178 10 studies with sample sizes of at least 40 participants and we anticipate considerable statistical
179 heterogeneity I^2=50%, with an estimated effect size of 0.35. Based on the aforementioned parameters
180 the estimated power for a fixed effects model is 0.93 and a random effects model is 0.69.

181 **Assessment of risk of bias in included studies**

For peer review only - http://bmjopen.bmj.com/site/about/guidelines.xhtml

Two review authors will assess independently the risk of bias using Cochrane's 'Risk of bias' tool for assessing risk of bias in each included study according to the following domains selection bias, performance bias, detection bias, attrition bias and selective outcome reporting bias [6]. We will resolve disagreements by discussion. We will specifically consider and report on the following sources of bias. We will grade each domain as low risk of bias, high risk of bias or unclear (lack of information or uncertainty of potential for bias). We will attempt to contact trial investigators for clarification of parameters graded as 'unclear'.

Dealing with missing data

If possible, we will conduct an intention-to-treat (ITT) analysis. We will use imputed data if computed by the trial investigators using an appropriate method but will not impute missing data ourselves. If ITT data are not available, we will do an available case analysis. This assumes that data are missing at random. We will assess whether this assumption is reasonable by collecting data from each included trial on the number of participants excluded or lost to follow-up and reasons for loss to follow-up by treatment group, if reported.

Assessment of heterogeneity and subgroup analysis

We will examine the overall characteristics of the studies, in particular the type of participants and types of interventions, to assess the extent to which the studies are similar enough to make pooling study results sensible. We will look at the forest plots of study results to see how consistent the results of the studies are, in particular looking at the size and direction of effects. We will calculate I^2 which is the percentage of the variability in effect estimates that is due to heterogeneity rather than sampling error. We will consider I^2 values over 50% to indicate substantial inconsistency but will also consider Chi^2 P value. As this may have low power when the number of studies are few we will consider $P < 0.1$ to indicate statistical significance of the Chi^2 test. If there are sufficient trials we will compare the effect of treatment in the following subgroups; diffractive, refractive and hybrid multifocal IOL and year of market approval.

Sensitivity analysis and assessment of reporting biases

For peer review only

For peer review only - http://bmjopen.bmj.com/site/about/guidelines.xhtml

We will examine the impact of excluding studies at high risk of bias in one or more domains. If there are 10 trials or more included in a meta-analysis, we will construct funnel plots and consider tests for asymmetry for assessment of publication bias, according to Chapter 8 of the Cochrane Handbook for Systematic Reviews of Interventions [7].

Limitations of this study

Bias such as lack of masking and confounding factors in the studies included will affect the certainty of the estimate of effect in our study. We will aim to mitigate against this by conducting sensitivity analysis by assessing the effect of excluding low quality studies. High heterogeneity amongst studies would reduce the power of this review. One of the reasons for this could be the use of different tools to measure the prevalence and extent of optical aberrations. Understanding whether the heterogeneity is clinical or statistical will be important and, in some instances, pooling of the data in a meta-analysis may not be appropriate. Publication bias could lead to overestimation of the true effect size, so clinical trial registries will be searched to identify unpublished results where possible. Furthermore, industry sponsored studies with conflicts of interests amongst investigators could introduce bias which would need to be evaluated.

Patient and Public Involvement

Patients and public were not involved in the development of this protocol. The primary outcome of the review is patient centered.

Ethics and Dissemination Ethics approval is not required for this review, as it will only include published data. Findings will be published in a peer-reviewed journal and disseminated across ophthalmic networks. We anticipate that the findings of this work will be of interest to multiple stakeholders: people who have undergone cataract surgery, eye health professionals, ophthalmic surgeons, device manufacturers and policy makers. It will also inform researchers to where there are gaps in evidence and identify areas for future research.

Authors contribution

For peer review only

For peer review only - http://bmjopen.bmj.com/site/about/guidelines.xhtml

MN conceived the idea for the review. CH, MK, AA and DB drafted and revised the protocol with suggestions from YB, CEHF and MN who reviewed the protocol and provided feedback on the draft. DB constructed the search.

Conflicts of interest

The authors declare no conflict of interest.

Funding

CH receives support from the National Institute for Health Research CL 2020-18-009

Data sharing statement

No additional data is available.

References

1. Salerno, L.C., M.C. Tiveron, Jr., and J.L. Alió, *Multifocal intraocular lenses: Types, outcomes, complications and how to solve them.* Taiwan journal of ophthalmology, 2017. **7**(4): p. 179-184.
2. Khandelwal, S.S., et al., *Effectiveness of multifocal and monofocal intraocular lenses for cataract surgery and lens replacement: a systematic review and meta-analysis.* Graefe's Archive for Clinical and Experimental Ophthalmology, 2018. **257**: p. 863-875.
3. de Silva, S.R., et al., *Multifocal versus monofocal intraocular lenses after cataract extraction.* Cochrane Database Syst Rev, 2016. **12**(12): p. Cd003169.
4. X. Wan, W.W., J. Liu, T. Tong, *Estimating the sample mean and standard deviation from the sample size, median, range and/or interquartile range.* BMC Med Res Methodol, 2014: p. 135.
5. Griffin, J.W., *Calculating statistical power for meta-analysis using metapower.* The Quantitative Methods for Psychology, 2021. **17**(1): p. 24-39.
6. Higgins, J.P., et al., *The Cochrane Collaboration's tool for assessing risk of bias in randomised trials.* Bmj, 2011. **343**: p. d5928.
7. Higgins JPT, T.J., Chandler J, Cumpston M, Li T, Page MJ, Welch VA (editors), *Cochrane Handbook for Systematic Reviews of Interventions version 6.2 (updated February 2021).* 2021.

For peer review only

Appendix 1. Search strategy

Ovid Medline 1946 – March 2021

Search Lines	Search Terms	Search Results
1	exp "Optics and Photonics"/	79373
2	(optic* or photonic*).mp.	476463
3	1 or 2	518769
4	exp Refractive Errors/ or exp Refraction, Ocular/ or exp Astigmatism/ or exp Myopia/ or exp Visual Acuity/	108238
5	(aberrat* or diffract* or refract* or HOA).mp.	461904
6	4 or 5	541199
7	exp Lenses, Intraocular/	15452
8	(intraocular lens or Intra-ocular lens or intra ocular lens or IOL or IOLs or lens prosthes* or artificial lens).mp.	18494
9	7 or 8	23720
10	(multifocal or multi focal or multi-focal or bifocal or bi-focal or trifocal or tri-focal or hybrid).mp.	218357
11	3 and 6 and 9 and 10	515

For peer review only

PRISMA-P (Preferred Reporting Items for Systematic review and Meta-Analysis Protocols) 2015 checklist: recommended items to address in a systematic review protocol*

Section and topic	Item No	Checklist item	Page Line
**ADMINISTRATIVE INFORMATION**			
Title:			
Identification	1a	Identify the report as a protocol of a systematic review	P1 L1
Update	1b	If the protocol is for an update of a previous systematic review, identify as such	P1 L1
Registration	2	If registered, provide the name of the registry (such as PROSPERO) and registration number	P3 L62
Authors:			
Contact	3a	Provide name, institutional affiliation, e-mail address of all protocol authors; provide physical mailing address of corresponding author	P2 L39
Contributions	3b	Describe contributions of protocol authors and identify the guarantor of the review	P1 L23
Amendments	4	If the protocol represents an amendment of a previously completed or published protocol, identify as such and list changes; otherwise, state plan for documenting important protocol amendments	NA
Support:			
Sources	5a	Indicate sources of financial or other support for the review	P2 L35
Sponsor	5b	Provide name for the review funder and/or sponsor	P2 L35
Role of sponsor or funder	5c	Describe roles of funder(s), sponsor(s), and/or institution(s), if any, in developing the protocol	NA
**INTRODUCTION**			
Rationale	6	Describe the rationale for the review in the context of what is already known	P4 L76
Objectives	7	Provide an explicit statement of the question(s) the review will address with reference to participants, interventions, comparators, and outcomes (PICO)	P4 L87
**METHODS**			
Eligibility criteria	8	Specify the study characteristics (such as PICO, study design, setting, time frame) and report characteristics (such as years considered, language, publication status) to be used as criteria for eligibility for the review	P4 L90
Information sources	9	Describe all intended information sources (such as electronic databases, contact with study authors, trial registers or other grey literature sources) with planned dates of coverage	P6 L122
Search strategy	10	Present draft of search strategy to be used for at least one electronic database, including planned limits, such that it could be repeated	P6 L22

For peer review only

Study records:			
Data management	11a	Describe the mechanism(s) that will be used to manage records and data throughout the review	P6 L136
Selection process	11b	State the process that will be used for selecting studies (such as two independent reviewers) through each phase of the review (that is, screening, eligibility and inclusion in meta-analysis)	P6 L132
Data collection process	11c	Describe planned method of extracting data from reports (such as piloting forms, done independently, in duplicate), any processes for obtaining and confirming data from investigators	P6 137
Data items	12	List and define all variables for which data will be sought (such as PICO items, funding sources), any pre-planned data assumptions and simplifications	P6 L141
Outcomes and prioritization	13	List and define all outcomes for which data will be sought, including prioritization of main and additional outcomes, with rationale	P5 L105
Risk of bias in individual studies	14	Describe anticipated methods for assessing risk of bias of individual studies, including whether this will be done at the outcome or study level, or both; state how this information will be used in data synthesis	P8 L147
Data synthesis	15a	Describe criteria under which study data will be quantitatively synthesised	P7 L154
	15b	If data are appropriate for quantitative synthesis, describe planned summary measures, methods of handling data and methods of combining data from studies, including any planned exploration of consistency (such as I^2, Kendall's τ)	P7 L154
	15c	Describe any proposed additional analyses (such as sensitivity or subgroup analyses, meta-regression)	P9 L208
	15d	If quantitative synthesis is not appropriate, describe the type of summary planned	P7 L159
Meta-bias(es)	16	Specify any planned assessment of meta-bias(es) (such as publication bias across studies, selective reporting within studies)	P9 L213
Confidence in cumulative evidence	17	Describe how the strength of the body of evidence will be assessed (such as GRADE)	P9 L217

*** It is strongly recommended that this checklist be read in conjunction with the PRISMA-P Explanation and Elaboration (cite when available) for important clarification on the items. Amendments to a review protocol should be tracked and dated.** **The copyright for PRISMA-P (including checklist) is held by the PRISMA-P Group and is distributed under a Creative Commons Attribution Licence 4.0.**

From: Shamseer L, Moher D, Clarke M, Ghersi D, Liberati A, Petticrew M, Shekelle P, Stewart L, PRISMA-P Group. Preferred reporting items for systematic review and meta-analysis protocols (PRISMA-P) 2015: elaboration and explanation. BMJ. 2015 Jan 2;349(jan02 1):g7647.

For peer review only

BMJ Open

Optical aberrations following implantation of multifocal intraocular lenses: a systematic review and meta-analysis protocol

Journal:	*BMJ Open*
Manuscript ID	bmjopen-2021-059350.R2
Article Type:	Protocol
Date Submitted by the Author:	25-Jul-2022
Complete List of Authors:	Henein, Christin; University College London Institute of Ophthalmology; NIHR Moorfields Biomedical Research Centre<br>Fang, Clarissa E H; Manchester Royal Eye Hospital<br>Bokre, Desta; University College London Institute of Ophthalmology<br>Khan, Maaz; University College London Institute of Ophthalmology<br>Adan, Ahmed; University College London Institute of Ophthalmology<br>Bouremel, Yann; University College London Institute of Ophthalmology<br>Nanavaty, Mayank; Sussex Eye Hospital; Brighton and Sussex Medical School
**Primary Subject Heading**:	Ophthalmology
Secondary Subject Heading:	Surgery, Evidence based practice, Ophthalmology
Keywords:	Cataract and refractive surgery < OPHTHALMOLOGY, Ophthalmology < SURGERY, OPHTHALMOLOGY

SCHOLARONE™
Manuscripts

Optical aberrations following implantation of multifocal intraocular lenses: a systematic review and meta-analysis protocol

Authors:

Christin Henein*[1,2], Clarissa E.H. Fang[3], Desta Bokre[1], Maaz Khan[1], Ahmed Adan[1], Yann Bouremel[1], Mayank A. Nanavaty[4,5]

*** Correspondence to Dr Christin Henein; c.henein@ucl.ac.uk**

Affiliations

[1]UCL, Institute of Ophthalmology, London, UK
[2]NIHR Moorfields Biomedical Research Centre, London, UK
[3]Manchester Royal Eye Hospital, Manchester, UK
[4]Sussex Eye Hospital, University Hospitals Sussex NHS Foundation Trust, Eastern Road, Brighton, United Kingdom. BN2 5BF
[5]Brighton & Sussex Medical School, University of Sussex, Falmer, Brighton. United Kingdom. BN1 9PX

Corresponding author*

I, the Submitting Author has the right to grant and does grant on behalf of all authors of the Work (as defined in the below author licence), an exclusive licence and/or a non-exclusive licence for contributions from authors who are: i) UK Crown employees; ii) where BMJ has agreed a CC-BY licence shall apply, and/or iii) in accordance with the terms applicable for US Federal Government officers or employees acting as part of their official duties; on a worldwide, perpetual, irrevocable, royalty-free basis to BMJ Publishing Group Ltd ("BMJ") its licensees and where the relevant Journal is co-owned by BMJ to the co-owners of the Journal, to publish the Work in BMJ Open and any other BMJ products and to exploit all rights, as set out in our licence.

Email addresses:

c.henein@ucl.ac.uk, d.bokre@ucl.ac.uk, maaz.khan.20@ucl.ac.uk, ahmed.adan.20@ucl.ac.uk, y.bouremel@ucl.ac.uk, fangclarissa@gmail.com, mayank.nanavaty@nhs.net

For peer review only

For peer review only

For peer review only - http://bmjopen.bmj.com/site/about/guidelines.xhtml

Abstract

Introduction: Multifocal IOLs are used to restore vision at different focal distances. The technology of multifocal IOLs is continually advancing. Optical aberrations a property of lenses that causes spreading of light over a region resulting in a blurred or distorted image. This study aims to systematically review investigator measured and patient reported optical aberrations following implantation of multifocal intraocular lenses during phacoemulsification surgery to treat presbyopia in adults.

Methods and Analysis: We will conduct an electronic database search for randomized controlled trials, prospective non-randomized studies, observational studies in Ovid MEDLINE, Ovid EMBASE, Cochrane Central Register of Controlled Trials (CENTRAL), Web of Science, Scopus, and ClinicalTrials.gov in March 2021. Eligibility criteria will include quantitative articles written in English and containing data on optical aberrations. Two independent reviewers will screen titles and abstracts and extract data from full texts, reporting outcomes according to Preferred Reporting Items for Systematic Reviews and Meta-Analyses (PRISMA) guidelines. Data extraction of key characteristics will be completed using customized forms. Methodological quality will be assessed using Cochrane Handbook 6.2.

Ethics and Dissemination Ethics approval is not required for this review, as it will only include published data. Findings will be published in a peer-reviewed journal and disseminated across ophthalmic networks. We anticipate that the findings of this work will be of interest to multiple stakeholders: people who have undergone cataract surgery, eye health professionals, ophthalmic surgeons, device manufacturers and policy makers. It will also inform researchers to where there are gaps in evidence and identify areas for future research.

Systematic review registration number: PROSPERO CRD42021271050

Keywords: Optics, aberration, intraocular lens, multifocal

Article Summary

For peer review only

Strengths and Limitations

- This systematic review protocol follows the Preferred Reporting Items for Systematic Review and Meta-Analysis Protocols guidelines.
- This systematic review addresses a gap in the current evidence-base by providing a comprehensive assessment of reported optical aberrations following new and older generation multifocal IOL
- There may be a paucity of RCTs comparing different multifocal IOLs limiting the number of paired wise meta-analysis that can be done.

Word count: 1507

Introduction

Multifocal intraocular lens (IOLs) have multiple focal lengths; if they have 2 foci, they are called bifocal, three foci, they are trifocal. This enables the patient with a multifocal IOL to see both objects located at a distance, intermediate distance or near to them. They are three different mechanisms to achieve this: the technology can be refractive, diffractive or combined. Moreover, toric multifocal lens also help to correct the problem of astigmatism [1].

Traditional monofocal IOLs provide a single point of focus. A newer enhanced monofocals and extended depth-of-focus (EDOF) IOLs which creates a single elongated focal point to enhance the depth of focus. For the purposes of this study we will assess optical aberrations following the implantation of different types of multifocal IOL and will exclude enhanced monofocal IOL as a well as EDOF IOLs.

It is generally accepted multifocal IOLS are good for distance and near focal distances. According to the lens design they can be refractive, diffractive or combined. The technology of multifocal IOLs is continually advancing. Next generation IOLs include rotationally asymmetric segmented multifocal IOL, increase in the central area with the aim to improve reading acuity and improved pupil independence.

For peer review only - http://bmjopen.bmj.com/site/about/guidelines.xhtml

Optical aberrations a property of lenses that causes spreading of light over a region resulting in a blurred or distorted image. Optical aberrations can present as symptoms of glare, holes and stars. This symptoms may limit the patient satisfaction achieved with these IOLs and is therefore an important patient-centred outcomes to quantify. Spherical aberrations significantly contribute to quality of retinal image and subjective refraction. Optical aberrations can be reported subjectively using questionnaires or measured objectively by wavefront aberrometry analysis. Contrast sensitivity can be a more useful/ objective tool to assess visual function. Recent reviews that compared multifocal with monofocal IOLs reported outcomes on spectacle independence, visual acuity and quality of life [2, 3]. To our knowledge this is the first review comparing different multifocal IOLs with optical aberrations as the primary outcome.

Review aim: We aim to systematically review investigator measured and patient reported optical aberrations following implantation of multifocal intraocular lenses during phacoemulsification surgery to treat presbyopia.

Methods and analysis

Inclusion and exclusion criteria

Types of studies

We will include randomised controlled trials (RCTs) and non-randomised interventional studies (retrospective or prospective studies). Observational studies will allow us to provide real-world estimates of reported optical aberrations.

Types of participants

We will include adults undergoing cataract surgery and desiring correction for anticipated post-operative presbyopia. We will exclude studies with participants with history of laser refractive surgery.

Intervention(s)

For peer review only

For peer review only - http://bmjopen.bmj.com/site/about/guidelines.xhtml

We will include small incision cataract extraction and multifocal lens implantation. All types of refractive and diffractive multifocal lenses will be included in this review.

Comparator(s)

We will include multifocal intraocular lens or alternative type of multifocal IOL as comparators such as diffractive, refractive and hybrid technologies.

Outcomes

Primary outcome

- Participant reported optical aberrations such as but not limited to glare and halos.

Secondary outcomes

- Measured optical aberrations with wavefront analysis
- Contrast sensitivity as measured by validated test
- Spectacle independence as determined by the participant or as determined by the investigator
- Uncorrected near vision acuity
- Uncorrected distance vision acuity
- Uncorrected intermediate distance
- Mean spherical equivalent within ±0.5D
- % of eyes seeing 20/20 or better for distance
- % of eyes seeing 20/40 or better for distance
- % of eyes seeing J2 or better for near vision
- YAG laser capsulotomy rates

Search strategy

For peer review only

In collaboration with an information specialist a comprehensive search strategy will be performed using a combination of controlled vocabulary and free text terms. Searches will be conducted in Ovid MEDLINE, Ovid EMBASE, Cochrane Central Register of Controlled Trials (CENTRAL), Web of Science, Scopus and ClinicalTrials.gov bibliographic databases. Other relevant sources will be searched such as reference lists of existing systematic reviews of multifocal IOLs. Please see supplementary file 1 for strategy syntax for Ovid Medline 1946 – March 2021 electronic database. We will download references identified in searches (electronic database and additional searches) into Endnote X9 reference management software and remove duplicate abstracts.

Study selection

The screening process will be undertaken using Endnote X9. Two review authors will independently assess the titles and abstracts of records and exclude papers that do not meet eligibility criteria. We will obtain the full text of the remaining papers, and at least two authors will assess the papers against the inclusion criteria for the review to determine their eligibility for inclusion. Non-English language papers will be excluded. The review authors will resolve disagreements through mediation with a third reviewer.

Data extraction

Two review authors will extract data independently using Excel. We will pre-pilot the data extraction template. We will resolve discrepancies by discussion. Two attempts will be made to contact trial investigators for missing data. Data will be directly imported into Review Manager 5 (RevMan 5); and the accuracy of the data import will be checked by one author.

We will collect the following information on study characteristics:

- Study design: parallel group RCT/within-person RCT/one or both eyes reported
- Participants: country, total number of participants, age, sex, inclusion and exclusion criteria
- Intervention and comparator details: type of multifocal IOL, including number of people (eyes) randomised to each group

For peer review only

- Primary and secondary outcomes as measured and reported in the trials
- Length of follow-up
- Date of publication
- Date multifocal IOL received market approval (FDA PMA, CE mark)
- Sample size
- Funding and conflicts of interest
- Trial registration, if available

Data synthesis

We will pool data where there are at least two studies for a particular type of mIOL reporting the same outcome. We will use a random-effects model in RevMan 5. But if there are fewer than three trials in a comparison we will use a fixed-effect model. If there is inconsistency between individual study results such that a pooled result may not be a good summary of the individual trial results — for example, the effects are in different directions or $I^2 > 50\%$ and $P < 0.1$ — we will not pool the data but will describe the pattern of the individual study results. If there is statistical heterogeneity we may pool the data if all the effect estimates are in the same direction, such that a pooled estimate would seem to provide a good summary of the individual trial results.

We will extract the following data from each included study for intervention and comparator groups separately.

- Number of events and number of participants on which outcome data collected for dichotomous variables
- Mean, standard deviation and number of participants on which outcome measured for continuous variables

For multi-arm studies we will use data relevant to our intervention and comparator groups. If two groups contain relevant data we will combine groups using the calculator within RevMan 5. If standard

For peer review only

For peer review only - http://bmjopen.bmj.com/site/about/guidelines.xhtml

182 deviation is not available we will use information from confidence intervals and P values, where
183 possible, to estimate it, using the RevMan 5 calculator [4].

184 For the primary outcome a power calculation will made using *metapower* package in R (rstudio.com)
185 to calculate the statistical power for meta-analysis based on Cohen's *d [5]*. We expect to find at least
186 10 studies with sample sizes of at least 40 participants and we anticipate considerable statistical
187 heterogeneity I^2=50%, with an estimated effect size of 0.35. Based on the aforementioned parameters
188 the estimated power for a fixed effects model is 0.93 and a random effects model is 0.69.

189 **Assessment of risk of bias in included studies**

190 Two review authors will assess independently the risk of bias using Cochrane's 'Risk of bias' tool for
191 assessing risk of bias in each included study according to the following domains selection bias,
192 performance bias, detection bias, attrition bias and selective outcome reporting bias [6]. We will resolve
193 disagreements by discussion. We will specifically consider and report on the following sources of bias.
194 We will grade each domain as low risk of bias, high risk of bias or unclear (lack of information or
195 uncertainty of potential for bias). We will attempt to contact trial investigators for clarification of
196 parameters graded as 'unclear'.

197 **Dealing with missing data**

198 If possible, we will conduct an intention-to-treat (ITT) analysis. We will use imputed data if computed by
199 the trial investigators using an appropriate method but will not impute missing data ourselves. If ITT
200 data are not available, we will do an available case analysis. This assumes that data are missing at
201 random. We will assess whether this assumption is reasonable by collecting data from each included
202 trial on the number of participants excluded or lost to follow-up and reasons for loss to follow-up by
203 treatment group, if reported.

204 **Assessment of heterogeneity and subgroup analysis**

205 We will examine the overall characteristics of the studies, in particular the type of participants and types
206 of interventions, to assess the extent to which the studies are similar enough to make pooling study

For peer review only

For peer review only - http://bmjopen.bmj.com/site/about/guidelines.xhtml

results sensible. We will look at the forest plots of study results to see how consistent the results of the studies are, in particular looking at the size and direction of effects. We will calculate I^2 which is the percentage of the variability in effect estimates that is due to heterogeneity rather than sampling error. We will consider I^2 values over 50% to indicate substantial inconsistency but will also consider Chi^2 P value. As this may have low power when the number of studies are few we will consider $P < 0.1$ to indicate statistical significance of the Chi^2 test. If there are sufficient trials we will compare the effect of treatment in the following subgroups; diffractive, refractive and hybrid multifocal IOL and year of market approval.

Sensitivity analysis and assessment of reporting biases

We will examine the impact of excluding studies at high risk of bias in one or more domains. If there are 10 trials or more included in a meta-analysis, we will construct funnel plots and consider tests for asymmetry for assessment of publication bias, according to Chapter 8 of the Cochrane Handbook for Systematic Reviews of Interventions [7].

Limitations of this study

Bias such as lack of masking and confounding factors in the studies included will affect the certainty of the estimate of effect in our study. We will aim to mitigate against this by conducting sensitivity analysis by assessing the effect of excluding low quality studies. High heterogeneity amongst studies would reduce the power of this review. One of the reasons for this could be the use of different tools to measure the prevalence and extent of optical aberrations. Understanding whether the heterogeneity is clinical or statistical will be important and, in some instances, pooling of the data in a meta-analysis may not be appropriate. Publication bias could lead to overestimation of the true effect size, so clinical trial registries will be searched to identify unpublished results where possible. Furthermore, industry sponsored studies with conflicts of interests amongst investigators could introduce bias which would need to be evaluated.

Patient and Public Involvement

For peer review only

For peer review only - http://bmjopen.bmj.com/site/about/guidelines.xhtml

Patients and public were not involved in the development of this protocol. The primary outcome of the review is patient centered.

Ethics and Dissemination Ethics approval is not required for this review, as it will only include published data. Findings will be published in a peer-reviewed journal and disseminated across ophthalmic networks. We anticipate that the findings of this work will be of interest to multiple stakeholders: people who have undergone cataract surgery, eye health professionals, ophthalmic surgeons, device manufacturers and policy makers. It will also inform researchers to where there are gaps in evidence and identify areas for future research.

For peer review only

Authors contribution

MN conceived the idea for the review. CH, MK, AA and DB drafted and revised the protocol with suggestions from YB, CEHF and MN who reviewed the protocol and provided feedback on the draft. DB constructed the search.

Conflicts of interest

The authors declare no conflict of interest.

Funding

CH receives support from the National Institute for Health Research CL 2020-18-009

Data sharing statement

No additional data is available.

References

1. Salerno, L.C., M.C. Tiveron, Jr., and J.L. Alió, *Multifocal intraocular lenses: Types, outcomes, complications and how to solve them.* Taiwan journal of ophthalmology, 2017. **7**(4): p. 179-184.

2. Khandelwal, S.S., et al., *Effectiveness of multifocal and monofocal intraocular lenses for cataract surgery and lens replacement: a systematic review and meta-analysis.* Graefe's Archive for Clinical and Experimental Ophthalmology, 2018. **257**: p. 863-875.

3. de Silva, S.R., et al., *Multifocal versus monofocal intraocular lenses after cataract extraction.* Cochrane Database Syst Rev, 2016. **12**(12): p. Cd003169.

4. X. Wan, W.W., J. Liu, T. Tong, *Estimating the sample mean and standard deviation from the sample size, median, range and/or interquartile range.* BMC Med Res Methodol, 2014: p. 135.

5. Griffin, J.W., *Calculating statistical power for meta-analysis using metapower.* The Quantitative Methods for Psychology, 2021. **17**(1): p. 24-39.

6. Higgins, J.P., et al., *The Cochrane Collaboration's tool for assessing risk of bias in randomised trials.* Bmj, 2011. **343**: p. d5928.

7. Higgins JPT, T.J., Chandler J, Cumpston M, Li T, Page MJ, Welch VA (editors), *Cochrane Handbook for Systematic Reviews of Interventions version 6.2 (updated February 2021).* 2021.

For peer review only

Ovid Medline

Search Lines	Search Terms
1	exp "Optics and Photonics"/
2	(optic* or photonic*).mp.
3	1 or 2
4	exp Refractive Errors/ or exp Refraction, Ocular/ or exp Astigmatism/ or exp Myopia/ or exp Visual Acuity/
5	(aberrat* or diffract* or refract* or HOA).mp.
6	4 or 5
7	exp Lenses, Intraocular/
8	(intraocular lens or Intra-ocular lens or intra ocular lens or IOL or IOLs or lens prosthes* or artificial lens).mp.
9	7 or 8
10	(multifocal or multi focal or multi-focal or bifocal or bi-focal or trifocal or tri-focal or hybrid).mp.
11	3 and 6 and 9 and 10

Ovid Embase

Search Lines	Search Terms
1	exp optics/
2	(optic* or photonic*).mp
3	1 or 2
4	exp eye refraction/
5	(aberrat* or diffract* or refract* or HOA).mp
6	4 or 5
7	exp lens implant/
8	(intraocular lens or Intra-ocular lens or intra ocular lens or IOL or IOLs or lens prosthes* or artificial lens).mp.
9	7 or 8
10	(multifocal or multi focal or multi-focal or bifocal or bi-focal or trifocal or tri-focal or hybrid).mp
11	3 and 6 and 9 and 10

For peer review only

PRISMA-P (Preferred Reporting Items for Systematic review and Meta-Analysis Protocols) 2015 checklist: recommended items to address in a systematic review protocol*

Section and topic	Item No	Checklist item	Page Line
**ADMINISTRATIVE INFORMATION**			
Title:			
Identification	1a	Identify the report as a protocol of a systematic review	P1 L1
Update	1b	If the protocol is for an update of a previous systematic review, identify as such	P1 L1
Registration	2	If registered, provide the name of the registry (such as PROSPERO) and registration number	P3 L62
Authors:			
Contact	3a	Provide name, institutional affiliation, e-mail address of all protocol authors; provide physical mailing address of corresponding author	P2 L39
Contributions	3b	Describe contributions of protocol authors and identify the guarantor of the review	P1 L23
Amendments	4	If the protocol represents an amendment of a previously completed or published protocol, identify as such and list changes; otherwise, state plan for documenting important protocol amendments	NA
Support:			
Sources	5a	Indicate sources of financial or other support for the review	P2 L35
Sponsor	5b	Provide name for the review funder and/or sponsor	P2 L35
Role of sponsor or funder	5c	Describe roles of funder(s), sponsor(s), and/or institution(s), if any, in developing the protocol	NA
**INTRODUCTION**			
Rationale	6	Describe the rationale for the review in the context of what is already known	P4 L76
Objectives	7	Provide an explicit statement of the question(s) the review will address with reference to participants, interventions, comparators, and outcomes (PICO)	P4 L87
**METHODS**			
Eligibility criteria	8	Specify the study characteristics (such as PICO, study design, setting, time frame) and report characteristics (such as years considered, language, publication status) to be used as criteria for eligibility for the review	P4 L90
Information sources	9	Describe all intended information sources (such as electronic databases, contact with study authors, trial registers or other grey literature sources) with planned dates of coverage	P6 L122
Search strategy	10	Present draft of search strategy to be used for at least one electronic database, including planned limits, such that it could be repeated	P6 L22

For peer review only

Study records:			
Data management	11a	Describe the mechanism(s) that will be used to manage records and data throughout the review	P6 L136
Selection process	11b	State the process that will be used for selecting studies (such as two independent reviewers) through each phase of the review (that is, screening, eligibility and inclusion in meta-analysis)	P6 L132
Data collection process	11c	Describe planned method of extracting data from reports (such as piloting forms, done independently, in duplicate), any processes for obtaining and confirming data from investigators	P6 137
Data items	12	List and define all variables for which data will be sought (such as PICO items, funding sources), any pre-planned data assumptions and simplifications	P6 L141
Outcomes and prioritization	13	List and define all outcomes for which data will be sought, including prioritization of main and additional outcomes, with rationale	P5 L105
Risk of bias in individual studies	14	Describe anticipated methods for assessing risk of bias of individual studies, including whether this will be done at the outcome or study level, or both; state how this information will be used in data synthesis	P8 L147
Data synthesis	15a	Describe criteria under which study data will be quantitatively synthesised	P7 L154
	15b	If data are appropriate for quantitative synthesis, describe planned summary measures, methods of handling data and methods of combining data from studies, including any planned exploration of consistency (such as I^2, Kendall's τ)	P7 L154
	15c	Describe any proposed additional analyses (such as sensitivity or subgroup analyses, meta-regression)	P9 L208
	15d	If quantitative synthesis is not appropriate, describe the type of summary planned	P7 L159
Meta-bias(es)	16	Specify any planned assessment of meta-bias(es) (such as publication bias across studies, selective reporting within studies)	P9 L213
Confidence in cumulative evidence	17	Describe how the strength of the body of evidence will be assessed (such as GRADE)	P9 L217

*** It is strongly recommended that this checklist be read in conjunction with the PRISMA-P Explanation and Elaboration (cite when available) for important clarification on the items. Amendments to a review protocol should be tracked and dated.** **The copyright for PRISMA-P (including checklist) is held by the PRISMA-P Group and is distributed under a Creative Commons Attribution Licence 4.0.**

From: Shamseer L, Moher D, Clarke M, Ghersi D, Liberati A, Petticrew M, Shekelle P, Stewart L, PRISMA-P Group. Preferred reporting items for systematic review and meta-analysis protocols (PRISMA-P) 2015: elaboration and explanation. BMJ. 2015 Jan 2;349(jan02 1):g7647.

For peer review only
